# ‘As soon as the umbilical cord gets off, the child ceases to be called a newborn’: sociocultural beliefs and newborn referral in rural Uganda

**DOI:** 10.3402/gha.v8.24386

**Published:** 2015-03-31

**Authors:** Christine K. Nalwadda, Peter Waiswa, David Guwatudde, Kate Kerber, Stefan Peterson, Juliet Kiguli

**Affiliations:** 1 School of Public Health, College of Health Sciences, Makerere University, Kampala, Uganda; 2 Health System Policy, Department of Public Health Sciences, Karolinska Institutet, Stockholm, Sweden; 3 Uganda Newborn Study, Iganga-Mayuge Health Demographic Surveillance Site, Kampala, Uganda; 4 Saving Newborn Lives, Save the Children, Cape Town, South Africa; 5 International Maternal and Child Health Unit, Department of Women and Children Health, Uppsala University, Uppsala, Sweden

**Keywords:** care-seeking, newborn, neonatal, qualitative, referral, sociocultural influences, Uganda

## Abstract

**Background:**

The first week of life is the time of greatest risk of death and disability, and is also associated with many traditional beliefs and practices. Identifying sick newborns in the community and referring them to health facilities is a key strategy to reduce deaths. Although a growing area of interest, there remains a lack of data on the role of sociocultural norms and practices on newborn healthcare-seeking in sub-Saharan Africa and the extent to which these norms can be modified.

**Objective:**

This study aimed to understand the community's perspective of potential sociocultural barriers and facilitators to compliance with newborn referral.

**Method:**

In this qualitative study, focus group discussions (n=12) were conducted with mothers and fathers of babies aged less than 3 months. In addition, in-depth interviews (n=11) were also held with traditional birth attendants and mothers who had been referred by community health workers to seek health-facility-based care. Participants were purposively selected from peri-urban and rural communities in two districts in eastern Uganda. Data were analysed using latent content analysis.

**Results:**

The community definition of a newborn varied, but this was most commonly defined by the period between birth and the umbilical cord stump falling off. During this period, newborns are perceived to be vulnerable to the environment and many mothers and their babies are kept in seclusion, although this practice may be changing. Sociocultural factors that influence compliance with newborn referrals to seek care emerged along three sub-themes: community understanding of the newborn period and cultural expectations; the role of community health actors; and caretaker knowledge, experience, and decision-making autonomy.

**Conclusion:**

In this setting, there is discrepancy between biomedical and community definitions of the newborn period. There were a number of sociocultural factors that could potentially affect compliance to newborn referral. The widely practised cultural seclusion period, knowledge about newborn sickness, individual experiences in households, perceived health system gaps, and decision-making processes were facilitators of or barriers to compliance with newborn referral. Designers of newborn interventions need to address locally existing cultural beliefs at the same time as they strengthen facility care.

The neonatal period is only 28 days and yet accounts for 44% of all deaths in children younger than the age of 5 years globally (1). In 2012, approximately 35,000 newborns in Uganda died during their first month of life (1). Three-quarters of these deaths occurred during the first 7 days of life (2), making the first week of life the most risky across the lifespan. The main causes of death during the newborn period are different from those in later childhood (3). More than any other age group, sick newborns can rapidly deteriorate and may die in a matter of hours. Yet, newborn health issues have only recently begun to receive specific attention in policy and programmes (4). Understanding local practices and beliefs during the first month and the first week are critical to optimise delivery of care and promote healthy behaviours to ensure newborn survival.[Fn FN0001]

A period of confinement or seclusion following delivery has been described in different settings, including Tanzania, Senegal, and Bangladesh, and is believed to protect the newborn from ancestral spirits and bad omens as well as infections and the cold (5–7). This seclusion period may pose a barrier to seeking care. Recognising illness and making a decision to seek care was identified as the main avoidable factor in 50% of newborn deaths in one study in eastern Uganda (8). The World Health Organization currently recommends home visits during the first week of life (9) to improve newborn survival (9). Depending on national policy, home visit activities range from preventive and promotive care to curative care. In addition, counselling, assessing for danger signs and referring sick newborns for health-facility-based care, is conducted.

The Uganda Newborn Study (UNEST) is a cluster randomised controlled trial designed to test a community-based care package for maternal and newborn health, including home visits by community health workers (CHWs) during pregnancy and the first week of life (4). While newborn-care practices have been evaluated in UNEST (10, 11), there is little data on the importance of local sociocultural norms and practices related to timely care seeking. This qualitative research sought to understand the community's perspective of potential cultural barriers and facilitators to compliance with newborn referral. This paper is the eighth in a series on UNEST.

## Methods

### Study setting and design

The study was conducted in Iganga and Mayuge districts in eastern Uganda, within the Iganga–Mayuge Health Demographic Surveillance Site (HDSS) (12). The HDSS consists of 65 villages that are predominantly rural. Thirteen of these villages are peri-urban and form Iganga Town Council. The HDSS population was 70,000 at the time of the study, with the main economic activity being subsistence farming. Other occupations include small-scale businesses such as grain milling, market vending, motorcycle transport, and civil service employment. The predominant ethnic group in the HDSS is the Basoga, whose indigenous language is Lusoga. The HDSS is served by one 100-bed hospital, six health facilities offering out- and in-patient services, and seven outpatient-only facilities (13). In Uganda, CHWs are integrated into national Village Health Teams which serve as volunteers at village level (14).

Within UNEST, locally recruited CHWs worked in their respective villages and visited households twice during pregnancy and thrice during the first week after birth, on days 1, 3, and 7, for counselling on health behaviours and danger signs, and newborn assessments (11). The CHWs were trained and expected to identify the following signs of severe illness: convulsion, hotness or coldness, failure to breastfeed, rapid breathing in a calm baby, grunting, lack of movement on stimulation, yellowing, in-drawing of chest, umbilical cord with pus, and skin papules with pus, with special attention to babies who were born preterm and/or of low birth weight. During the home visit, the CHWs advised the mothers to seek care from a health facility immediately if a danger sign was noticed. In addition, CHWs also referred babies born outside health facilities for immunisation and postnatal care. In all the referral cases, a CHW wrote a referral note and gave it to the mother to present at the health facility for faster assistance than if they were to seek care without a referral.

This qualitative study was conducted between January and September 2012. Homogeneous focus group discussions (FGDs) and in-depth interviews (IDIs) with mothers, fathers, and traditional birth attendants (TBAs) were used to explore the community's perspectives in terms of knowledge of norms, perceptions, and practices during the newborn period which might impact on danger sign recognition, care-seeking practices, and decision-making processes, as well as referral compliance. The TBAs were considered important informants with unique perspectives on newborns and healthcare-seeking, since many women in the study area seek care from traditional healthcare providers, even if they do end up delivering at the health facility.

The field research team comprised a public health specialist and a medical anthropologist with social scientists as research assistants.

### Sampling frame and sampling procedures

The selection of study participants was purposive, targeting community members believed to have rich information about sociocultural factors that could influence compliance with newborn referral advice. Eight rural and four peri-urban villages were randomly selected from the 65 villages in the study area to host the FGDs and IDIs. FGDs were categorised by geographical location, sex (male/female), age (18- to 25-year-old and 26- to 49-year-old females were split), and place of residence (rural/peri-urban). The mothers who participated in IDIs were further categorised according to compliance with referral (complied/did not comply). The rationale behind categorisation was to obtain maximum variation in the views of the study participants (15). Attributes such as geographical location and mother's characteristics have been documented as influencing utilisation of healthcare services (16).

### Recruitment of study participants

Male and female participants for both the FGDs and IDIs were purposively selected from the villages with the assistance of the local village head. Participants were required to have children aged less than 3 months in order to identify individuals with knowledge and experience of the current norms and practices surrounding newborn care in this setting. UNEST staff and local CHWs provided information used to identify the homes of mothers who participated in the IDIs. None of the persons invited for interviews or discussions declined to participate.

### Data collection and processing

Research assistants whose mother tongue is Lusoga and who were experienced in qualitative data collection methods participated in data collection either through note-taking or moderating the discussions. They were trained on the study objectives and familiarised with the discussion and interview guides. Two pilot FGDs and one IDI were conducted in a village neighbouring the study area to test the flow of the discussions and interviews and how long each would last. Thereafter, some questions were rephrased for clarity and others rearranged to facilitate logical flow of the discussion.

A total of 12 FGDs were conducted. Each FGD comprised 5–10 participants and lasted no longer than 2 h (17). In addition, IDIs were conducted with TBAs (n=3) and women who had a sick newborn and subsequently received a referral by a CHW to seek further care (n=8). This was done to triangulate the issues expressed during the FGDs and deepen understanding of the social and cultural factors influencing care-seeking.

The FGD guide was based on questions formulated according to themes from previous research findings, but remained flexible to address emerging issues during the discussions. IDI participants were asked similar introductory questions as in the FGDs, but were also asked and probed for information such as why they complied with referral or why they did not comply, and who was responsible for this decision making. In the case of TBAs, their role in caretakers’ compliance and decision making was also ascertained. The FGDs and IDIs were held at venues agreed upon by the participants and free from interruption such as from onlookers and traffic.

All interviews and discussions were conducted in Lusoga, the main local language in the study area. They were all tape-recorded and notes were taken. The note takers immediately expanded the notes after the interviews and discussions, in preparation for supplementing the recordings at a later date during the verbatim transcription in cases where recordings were not audible enough. Emerging issues were identified and explored further in subsequent interviews and discussions.

The research assistants provided verbatim transcriptions of the recordings with direct translation into English. Local terms that could not easily be translated into English were kept in Lusoga. The data were analysed using content analysis (18). The conventional content analysis approach was used, where codes are directly derived from the data text (19). The FGD and IDI scripts were independently read by three people: the principal investigator, a medical anthropologist, and a social scientist. These readings were used to generate codes that were discussed and agreed upon during a review meeting and used for data analysis. The principal investigator developed the final code book, closely guided by the medical anthropologist. The code book was accessible to all research team members.

The text data were systematically synthesised. First, they were coded into meaning units, which were grouped into categories, followed by sub-themes, and then the final themes as agreed upon by the study team. This analysis was led by the medical anthropologist. The ATLAS.ti version 7.0 software program was used for qualitative data management and analysis. Further development of meaningful units and interpretation was done by all of the authors. Disagreements were resolved through consensus discussions.

### Ethical considerations

The protocol for this study was reviewed and approved by Makerere University College of Health Sciences, School of Public Health Higher Degree Research Committee, and the Uganda National Council of Science and Technology. The research team members explained the purpose of the study to the participants and emphasised that enrolment into the study was voluntary. Individual written consent was sought from both FGD and IDI participants. Illiterate participants were verbally informed of the terms of the study and provided consent through thumbprints. Only participants aged 18 years and above were recruited for interviews and discussions. No money or incentives were offered to the study participants, although a soft drink was given to each participant during the discussions. Interviews and discussions took place in privacy, and to ensure confidentiality, FGD participants were assigned identification numbers during discussions and transcription. In addition, all files were password protected.

## Results

The sociodemographic profile of the participants is presented in Table 1. A total of 48 women participated in the eight FGDs and eight IDIs, with a mean age of 28.9 years and range of 18–44 years. The majority of participants were married (n=44), had attained primary education (n=34), and were engaged in subsistence farming (n=42). Three TBAs were interviewed, aged 60, 50, and 48 years. A total of 23 men participated in four men's FGDs, with a mean age of 39.7 years and range of 22–57 years. The mean number of children fathered by these participants was 5 with a range of 1–14. The men were mainly subsistence farmers (n=9); an equal number (n=9) had attained ordinary-level secondary education. Amongst the eight women interviewed for IDIs, four mothers had sought referral care for their newborns (complied), and four women had not.

**Table 1:** Sociodemographic characteristics of men and women who participated in FGDs and IDIs

Variables	Men N=23 n (%)	Women N=48 n (%)
District		
Iganga	12 (52)	16 (33)
Mayuge	11 (48)	32 (67)
Residence		
Peri-urban	10 (43)	14 (29)
Rural	13 (57)	34 (71)
Marital status		
Single/never married	5 (22)	4 (8)
Married	18 (78)	44 (92)
Education status^a^		
Primary	9 (39)	34 (71)
Secondary ordinary	6 (26)	8 (17)
Secondary advanced	2 (9)	1 (2)
Tertiary	3 (13)	0 (0)
No education	3 (13)	5 (10)
Occupation^b^		
Peasant	14 (61)	40 (83)
Businessman	6 (26)	4 (8)
Catering	0 (0)	1 (2)
Housewife	0 (0)	2 (4)
Tailor	0 (0)	1 (2)
Head teacher	1 (4)	0 (0)
Primary teacher college tutor	1 (4)	0 (0)
Student	1 (4)	0 (0)

a Years of formal education: primary (1–7 years), ordinary-level secondary (8–11 years), advanced level secondary (12–13 years), and tertiary (14+ years).

b Categories for men and women do not add up to 100% due to rounding off of the values.

The results of the FGDs and IDIs revealed three broad themes that may influence care-seeking and compliance with referral: community understanding of the newborn period and cultural expectations; the complexity of community health actors; and caregiver knowledge, experience and decision-making autonomy (Table 2).

**Table 2:** Qualitative data analysis of the FGD and IDI feedback transcribed verbatim

Text/verbatim extract	Meaning unit	Condensed unit	Sub-theme	Themes
We call the baby ‘nakaghwele’. ‘Nakaghwele’ is the newborn and ‘omwibo’ is the mother. Because she is still in the postnatal period ‘akali mwibo’	‘Nakaghwele’ is the newborn and ‘omwibo’ is the newly delivered mother	Cultural terms for newborn and mother	Newborn and mother cultural terms	Understanding newborn definition
‘Nakaghwele’ starts at 1 day up to: 6 weeks …, 3 months …, 6 months when he/she starts eating and drinking …, starts to crawl/walk …, ends at 2 years	Newborn period defined in various ways	Varying definitions of newborn in community	No common newborn definition	Understanding newborn definition
Now, we the Basogas have cultural norms …, A baby with an umbilical cord does not come out of the house and doesn't cross the road unless when sick	A baby with an umbilical cord does not come out of the house	Newborn kept in house till umbilical cord detaches	Seclusion period	Understanding newborn period and cultural expectation
It's to all, irrespective of boy or girl; the cord must go off before he/she is brought out of the house	Both female and male newborns are kept inside until cord detaches	Male and female babies treated the same	Seclusion norm – no differentiation by sex	Understanding newborn period and cultural expectation
If Sande (CHW) gives me a referral form, I don't line up; when I reach there, I just show them the referral form and they will work on me; you don't have to wait like the one without a referral form	Mothers referred by CHWs with a referral note are attended to quickly at health facilities	CHWs’ referrals recognised by health workers	CHWs link mothers to health facilities	Role of community health actors
No, we go there because clinics are near and easy to access. And still it depends on the baby's situation; you may just go and get first aid from clinics before you proceed to the hospital	Seek care from private clinics due to easy access and for first aid	Seek care from private clinics	Private clinics offer services	Role of community health actors
There are times when you give birth to a child and he/she develops what they call ‘bidama’ in Lusoga, you have to take the child for traditional intervention	Local/traditional diseases are treated at traditional healers	Traditional illness requires traditional treatment	Care from traditional sector	Role of community health actors
At Musawo Monica's clinic in the trading centre and that is where she was immunised from because they send vaccines from Magada every end of the month	Mothers are aware and immunise children at outreaches	Private clinics used for immunisation outreaches	Public–private partnership	Role of community health actors
When the body changes and becomes yellow, child has a problem …, if the baby has been breastfeeding well, the feeding pattern reduces or it cannot suckle	Baby has a problem when he/she turns yellow …, if he/she reduces or stops breastfeeding	Yellowing, failure to breastfeed show baby is unwell	Knowledge of danger signs	Caregiver knowledge
Sometimes if the past experience from Nakavule (Hospital) was very bad so you are forced to go to clinics	Go to clinics due to bad experience at hospital	Bad experience at hospital	Past experience at health facility	Caregiver experience
When you are a husband at home, yet you don't have money, the one who has money decides	The one who has money is the one who decides	Decision depends on availability of money	Both mother and father can decide	Decision-making autonomy

### Community understanding of the newborn period and cultural expectations

All the FGD and IDI participants mentioned and agreed that the local term for a newborn in this setting is *nakaghele* while the mother is known as *omwibo*, meaning ‘one who has just delivered’. There was no single common definition of the newborn period. Few respondents conveyed the epidemiological definition, that is, from birth through 28 days of life. Rather, often participants described different time-points as marking the end of the newborn period. These included commencement of supplementary feeding, attainment of specific growth milestones such as walking and ability to care for self. For example, one woman in the FGD said ‘A newborn can go up to 6 months because that is when the child starts eating and drinking’, while a man in another FGD said that ‘a newborn stops at 8 months because when my child starts walking it has come out of newborn size’.

The trained CHWs reportedly provided a consistent definition of the newborn period as that from birth to 1 month of age, as narrated by an informant during an interview: ‘The village health worker (*mentions the name of the CHW*) said a newborn is a 1-month-old baby’. However, the community's most common indicator of the end of the newborn period was umbilical cord detachment. The participants frequently reported that the umbilical cord detachment took place within a week after delivery, and this marked the end of the newborn period, as explained by one male FGD participant:

A child ‘nakaghwere’ [*the young one*] is the one who is kept in the house and the cord has not yet got off …, when it gets out of the house it stops being a newborn after 7 days, then it ceases to be a newborn. It is our cultural practice, as long as the cord gets off it ceases to be called a newborn.

In many discussions and interviews, a newborn was consistently described as ‘a child who is delicate’ and needed extra care and protection, including shielding from sunshine, cold, and disease vectors such as mosquitoes and rats. One man in the FGD explained that ‘you cannot just hold the newborn anyhow, you must be very careful with extra care …, not putting the baby in the sun and cold …, keep baby away from insects, even rats …, laughter …’. In another FGD, a woman explained that ‘A newborn is defined from day 1 of birth up to 1 year because you have to breastfeed, care about him all the time … and if left to the cold, it might get this fever pneumonia that comes with the cold’.

One of the distinctive norms is the postnatal seclusion or confinement period known as *ekisanda* (dry banana leaves). The practice does not have a specific number of days but it begins immediately after birth and continues until the newborn's umbilical cord falls off. This was explained by one male FGD participant: ‘The mother and *nakaghwele* (newborn) when still in *ekisanda* (seclusion) cannot move out until after the umbilical cord drops off … and at that moment it will be called *omwana wawa* (a grown-up child)’.

Culturally, mothers are expected to strictly observe this seclusion period and stay indoors, irrespective of their place of delivery. However, this was reported to be changing, especially in the peri-urban areas, as explained by one informant: ‘For us here in town we can't stay in house without moving out, you have to go to the market and buy food every day, even take the baby if sick …’ (IDI mother who complied with referral advice).

A woman in one peri-urban FGD said that it was possible to violate the seclusion period: ‘But nowadays for me …. I have to take my baby to the hospital when sick, whether the umbilical cord still on or not’ (mother, FGD). Similarly, a man in the FGD held in the peri-urban area also reported that health workers instruct them to take babies for immunisation within the first week after delivery: ‘The health workers teach us to take the child to the health centre for polio prevention, even though it has not yet made 7 days’ (father, FGD).

Traditionally, during this period, a woman who has just delivered is supposed to sleep on the floor on dry banana leaves or on a mattress with a few dry bananas leaves underneath. During this period, the baby is not supposed to be touched by outsiders, not even siblings. This seclusion period is intended to protect the baby against evil spirits and infections, and the cold that may lead to sicknesses such as pneumonia. There does not appear to be a difference in gender regarding the period of seclusion, as mentioned by one informant: ‘Whether a boy or a girl, they are not taken out so long as the umbilical cord is not yet off’ (IDI mother who did not comply with referral advice).

However, several participants indicated that in the case of a critical situation (e.g. a funeral of a close family member), a woman could be allowed to attend the funeral and burial. Often participants made provisions to violate the confinement and seek healthcare for sick babies and those who needed routine immunisation. This was one practice reported that was in conflict with seclusion practices. However, a few participants reported the desire to strictly observe the seclusion period unless a misfortune happened, as one woman said: ‘I cannot go out before the umbilical cord drops off – maybe if my mother or father died’ (mother, FGD).

After the umbilical cord falls off, a number of rituals, *okukuza omwana* (recognising that the baby is a grown-up), are performed, such as the naming ceremony. Other practices include ritual bathing, throwing away the dry banana leaves from the seclusion room, parents of the newborn engaging in sexual intercourse for the first time since delivery (or a ritual to symbolise the same), and feasting, as participants described:

Ceremonies are performed after the baby's cord has dropped off. We call it *okukuza omwana*. The mother throws away the dry banana leaves, the father and mother of the baby sleep together for the first time after delivery, and depending on the clan of the family, different foodstuffs like sim-sim sauce and goat's meat are prepared for the family members to eat. (IDI, TBA)

When the baby's umbilical cord is off, [the mother and baby] move outside, pour water on the roof and it comes and falls on the child. (father, FGD)

The umbilical cord is kept secure by the mother or given to the father of the newborn. The umbilical cord is kept to be used in another ceremony of confirming paternity at a later date.

### The role of community health actors

Community care providers such as CHWs, private drug sellers, and TBAs play various roles with regard to maternal and newborn care. Two TBAs reported that they refer sick newborns and pregnant mothers who are first-time mothers and those who have had five or more deliveries, as one explained: ‘I once sent a mother to take the baby who had a cord with pus to Iganga Hospital; the mother had put powder on it’.

Many of the FGD participants reported that CHWs encourage community members to seek care from health facilities, and also agreed that families comply with referral advice given by the CHWs, as mentioned by one of the women in the FGD: ‘Our village health worker told us that before we buy drugs for the baby from shops, we should first take them to Government health centres. So we also follow her advice’.

The participants continued to mention that CHWs offered them health education about newborn care and motivated them to seek referral care by giving them referral notes, as one woman in the FGD stated: ‘When the CHWs are teaching us, they tell us about the bad signs, when you realise that the baby has some of those bad signs, you go to the CHW …, the CHW gives you a note (*referral slip*) and you go to the health facility’.

However, for some community members, the first point of care-seeking following referral may not be the health facility, even when they have been counselled. One informant reported that some caretakers first seek care from drug shops and traditional providers, given their proximity, especially during the seclusion period. Depending on the baby's outcome after receiving initial care, they may decide to proceed to a health facility, as stated by a mother during an interview: ‘Some people first go to drug shops and traditional healers and then to the Government health facilities if the baby does not get well’.

Another reason given for not immediately seeking formal care following the CHWs’ referral advice was the belief that some conditions are best managed by traditional healers or community specialists. Compliance with referral can be based on whether the cause of the sickness is believed to have a traditional or biomedical solution. One mother stated during the interview: ‘… when the baby has a strange disease like the yellow body, you go to a traditional healer’ (IDI mother who did not comply with referral).

### Caretaker knowledge, experience, and decision-making autonomy

#### Caretaker knowledge of newborn danger signs

FGD and IDI participants often mentioned symptoms that indicate severe illness, such as failure to breastfeed, convulsions, and umbilical cord with pus. These symptoms are in agreement with those classified as newborn danger signs by biomedical health workers. However, signs which do not qualify as danger signs were occasionally mentioned by participants, and these included ‘false teeth’ (20), ‘extra digits’, and ‘tongue tie’. The participants agreed among themselves that newborns with danger signs should be taken for facility-based care. The community's knowledge of danger signs seemed to influence compliance with referral. Caregivers who could describe the danger signs promoted by CHWs were positively inclined towards complying and seeking referral care, as stated by one woman in the FGD: ‘A baby may have a problem with the umbilical cord, like you see blood or pus coming out of the cord. You go to the CHW if the CHW is able to help; she can help and thereafter can send you to a health facility’.

#### Caretaker experiences

During the FGDs and IDIs, participants mentioned and narrated negative experiences from previous interactions with the health system. These reflected poor quality of care at the health facilities, such as rude health workers, stock-outs of medicines, and absenteeism from work, especially on weekend days. These were highlighted as possible barriers to newborn referral compliance. Participants expressed the view that some health workers were rude, and thus discouraged caretakers from seeking referral care. It was also reported that health workers at lower-level facilities did not work during evening hours or weekends, so that if a newborn is referred during a weekend, the caretaker has few options. Similarly, perceived poor quality of care, including lack of drugs and supplies, deters caretakers from seeking a higher level of care, especially if there is much distance to the facility.

Some participants reported that there were drug stock-outs at health facilities and that they were told to buy medicine for their newborns. With such experiences, some caretakers may opt for other alternatives to treat the sick newborns. Acknowledging these constraints and perhaps reflecting pervasive beliefs in traditional remedies, health workers were also reported to have advised parents to seek care in the informal health sector, as one mother reported: ‘Sometimes health workers when they don't have that medicine they tell you to go back and (get) local medicine’ (mother, FGD).

Experiences with stock-outs in particular were raised as potential barriers to future compliance with referral. At the same time, positive experiences such as the presence of qualified personnel and high technology such as blood transfusion services were possible motivators to seeking care from health facilities, as expressed by one informant mother who complied with referral: ‘I took the baby to Nakauvle (district hospital) because there are doctors who can put in blood’.

Informants who did not comply with referral reported experiences related to the health status of the mother and family members, poverty, and weather conditions as factors that prevented them from complying with referral. One of the informants explained: ‘I was unwell, still bleeding and could not walk to take the baby’. Another mother said ‘I did not go because it had rained the whole day and I had no money for transport’, and another that ‘I was caring for another sick child. I decided to wait and go another day’.

#### Decision making

According to most FGD participants, the decision to seek referral care when recommended rested primarily with the father or mother rather than other family or community members. They were in agreement with a TBA who reported that as an outsider to the family, she was not responsible for making any decisions when the baby was referred by the CHWs; her role ends at delivering the babies. She explained: ‘When the CHW (*mentions CHW's name*) tells the mother to take the baby to Nakavule (Hospital), it is for the family to decide if they take the baby, not me – my job is to help the mother deliver the baby’.

Occasionally, some women expressed that while the mother can take a decision to seek care, she often does not have the finances to be able to do so. Other participants were of the view that since the father provides money, he ultimately makes the decision, as narrated by one woman in the FGD: ‘You a woman may decide to take the baby after being referred, but if you don't have the money you find yourself unable to go’. Another woman in the FGD added: ‘Now if the baby refuses to breastfeed or has a high temperature, I told you, our village health workers write letters for us and tell us to take the babies to the health facility, but it's for the husband to decide’. During the interview, another mother emphasised that: ‘He has to decide because he is the one to pay the hospital bills, so you cannot force him’ (IDI mother who did not comply with referral).

## Discussion

Sociocultural factors influencing community newborn referral compliance in eastern Uganda were broadly categorised into those related to the community understanding of the newborn period and expected practices around this time, the varying and overlapping roles of community health actors, and caretaker knowledge, experience, and decision making. These were the main themes that emerged linked to whether or not families comply with community referrals. In general, caretakers portrayed willingness to comply with newborn referrals, for example, from CHWs, but there were also barriers to their ability to seek care.

We found a discrepancy between the biomedical and community definitions of a newborn. While some of the study participants described the newborn period as lasting through the first year, the predominant definition limited the newborn period until the baby's umbilical cord stump fell off. The newborn period was linked to a number of strongly held traditional practices, including seclusion for mother and baby in some families, which may affect care and care-seeking. A seclusion period is well described in the literature from other settings (7, 21–23), although in this setting the time was shorter than the commonly held 40 days of life (5, 6). While this practice provides an opportunity for the mother to rest and regain strength, establish breastfeeding, and bond with her baby, it might also limit the ability to seek care outside of the home during the time of greatest risk (5, 6, 24). However, participants in this setting reported a possibility of breaking the seclusion and seeking referral care if the illness is severe enough. Participants expressed that the seclusion period is a traditional practice, but that if necessary the baby can be taken for facility-based care.

Recognition of danger signs and perceiving certain symptoms as important, eliciting seeking of care, may have been due to counselling by the CHWs. For example, a bleeding cord or one with pus was associated with the baby having severe illness, and prompted caretakers to comply with referrals. CHWs are recognised in their capacity as health promoters for women and newborns in this setting, a finding that has been also been echoed in western Uganda, where the integrated community case management programme for children under 5 years of age has been rolled out (25).

Both mothers and health workers noted that the referral slips given to women by CHWs to present at health facilities increase legitimacy and facilitate prompt treatment by the health workers (25). However, families do not always go directly to formal health services after referral. Traditional providers and local private clinics and drug sellers were more likely to be the first point of care, especially in rural areas and if the baby is referred in the evening or on weekends. This has implications for newborn survival, despite the increased care-seeking, since such places have limited capacity for and quality of sick newborn management, and may lead to delays in receiving appropriate care. Referrals from community level were not just given by CHWs, and the reported referral by TBAs is important emerging information that needs more study.

In one review of home-based newborn care in Bangladesh, the use of traditional care and home remedies was preferred to formal health services (26). Only when the baby did not improve at home did the caretaker consider seeking care from the health facilities. In Ghana, only 61% of newborns with a serious illness were taken outside the home to seek care (27). This phenomenon of seeking facility-based care after failure to improve has also been reported amongst older children in Tanzania (28). Informal healthcare providers play an important role in offering health care because of their proximity and availability in the communities. Involving them early on in community-based interventions should be explored, and it should also be examined how they can support care-seeking from the formal health sector for newborn illness and reduce delays in receiving newborn care.

Decisions to seek referral care ultimately depend on the availability of finances, which are usually controlled by the husband, who is most often the head of the household. In general we found that the source of the decision to seek care was mixed. In other families the father was viewed both as an overall decision-maker in the home in addition to providing the money. This is similar to a finding reported in a study that explored how decisions are made in seeking maternal care in Burkina Faso (29). The same opinion that men dominate in decision making at household level was reported by Mbizvo and Adamchak in Zimbabwe (30). However, we also found that mothers often believed that they had a right and that it was their role to make a decision in relation to the baby's health, although sometimes they may not have had the money to go for referral care.

Past experiences with seeking care from the formal sector and perceived quality of care seemed to have a role to play in compliance with the CHWs’ newborn referral advice. Past experience with drug stock-outs, health work absenteeism, and rudeness could lead to non-compliance in this setting, as reported by Waiswa et al. (31). This experience is not unfounded; previous studies show that families in Uganda may not receive high-quality care for their sick children once they do seek care (25, 32, 33). The health system quality gaps thus need to be overcome to further motivate caregivers to comply with referral advice.

This study has some limitations. Transferability of some of the findings may only apply to the study setting and areas with similar cultural norms. However, findings regarding decision making and health system gaps as barriers to compliance with newborn referral may be more widely applicable. The use of FGDs first followed by IDIs may not be considered the normal approach, but in this study we need to understand the broader picture before narrowing down to the participants who experienced the newborn referrals.

We aimed at achieving trustworthiness in this study by triangulation of the data collection methods, providing a description of quotes from the participants for accuracy of representation, describing the data analysis process to show that the findings emerged from the data, with the team comprising experienced researchers in the method and topic of study.

## Conclusion

In this setting a discrepancy exists between the biomedical and community definition of the newborn period. There were a number of sociocultural factors that could potentially affect compliance to newborn referral. Despite the widely practised norm of a seclusion period, acceptance to seek referral care during this period seems feasible, particularly if the illness in the newborn is perceived as severe. Social factors, including knowledge about newborn sickness, individual experiences at households, perceived health system gaps and decision making processes, were facilitators of or barriers to compliance with newborn referral.

Designers of interventions for newborns need to address locally existing cultural beliefs at the same time as they strengthen facility care. Additional emphasis on all danger signs should be made during CHW counselling sessions in pregnancy and the early postnatal period. The differences between illnesses with a perceived biomedical solution compared to those considered suitable for treatment by traditional remedies and the subsequent impact on care-seeking patterns requires more investigation. Similarly, more research is needed to identify factors that can reduce delays in care-seeking, including how to better engage trusted community health actors such as TBAs.

## Footnotes

§ Senior authors contributed equally.
